# Epidemiological Trends and Seasonal Patterns in Childhood Type 1 Diabetes: Insights From 2001 to 2024 in Lithuania

**DOI:** 10.1155/pedi/9968982

**Published:** 2026-03-06

**Authors:** Ingrida Stankute, Dale Marciulionyte, Rytas Ostrauskas, Aiste Cemerkaite, Gryte Leonaviciute, Robertas Kemezys, Sigita Vainiene, Kastytis Smigelskas, Rasa Verkauskiene

**Affiliations:** ^1^ Institute of Endocrinology, Medical Academy, Lithuanian University of Health Sciences, Kaunas, Lithuania, lsmuni.lt; ^2^ Institute of Microbiology and Virusology, Veterinary Academy, Lithuanian University of Health Sciences, Kaunas, Lithuania, lsmuni.lt; ^3^ Medical Academy, Lithuanian University of Health Sciences, Kaunas, Lithuania, lsmuni.lt; ^4^ Institute of Clinical Medicine, Faculty of Medicine, Vilnius University, Vilnius, Lithuania, vu.lt; ^5^ Klaipeda Children’s Hospital, Klaipeda, Lithuania, ku.lt

**Keywords:** children, epidemiology, incidence, seasonal variability, trends, type 1 diabetes

## Abstract

**Background:**

Type 1 diabetes (T1D) incidence is rising globally, with significant regional variation. Data from highly homogeneous populations, such as Lithuanian, may contribute to a better understanding of contributing T1D factors. This study examines 24‐year trends in childhood T1D incidence and seasonal patterns in Lithuania.

**Methods:**

The annual incidence rates (IRs) were computed utilizing established methodologies per 100,000 children <15 years. The study included 2369 (1181 boys) patients with T1D.

**Results:**

During 2001–2024, the mean IR was 21.4 per 100,000 < 15 years (95% CI: 20.89, 27.28). The incidence rose from 10.8 to 36.4 per 100,000 children under 15 years of age, with notable peaks observed in 2021 and 2022, temporally aligning with the highest COVID‐19 infection waves. Subgroup analysis showed the most rapid increase in young teenagers (10–14 years). Most new cases (63.5%) were diagnosed from September to March.

**Conclusions:**

This study demonstrates a rapidly increasing incidence of T1D in Lithuanian children over a 24‐year period and is one of the highest in European countries. The seasonal distribution of new cases has been speculated to be due to reduced sunlight exposure and lower vitamin D levels, as well as increased school related stress and viral infections during autumn and winter months. However, additional contributing factors are likely involved, underscoring the need for further research.

## 1. Introduction

Type 1 diabetes (T1D) is the most prevalent chronic non‐communicable disease diagnosed in children. According to the International Diabetes Federation (IDF) Atlas 11^th^ edition, over 1.8 million children and adolescents were living with T1D worldwide [1]. Moreover, it is reported that approximately 200,000 children and adolescents are diagnosed with T1D annually [1].

Though epidemiological data consistently indicate an upward trend in the incidence of childhood T1D worldwide, there is a substantial variability across different countries. The lowest incidence rates (IRs) have been reported in African and Western Pacific countries, while the highest rates are observed in Finland, moreover, some countries in Middle East (United Arab Emirates, Saudi Arabia, and Oman) have reported incidence almost equal to or even surpassing Finnish, respectively [1].

Regardless of extensive long‐term epidemiological studies, the definitive evidence regarding the underlying contributing factors for increasing IRs of T1D—whether genetic, environmental, or lifestyle‐related—remains limited [2]. Meanwhile, the rising prevalence of T1D undoubtedly imposes a substantial and growing economic burden on healthcare systems globally [2]. Conducting comprehensive research on the epidemiology of T1D in children, particularly in genetically and culturally homogeneous populations such as Lithuanians, could provide valuable insights into the etiological factors driving this increasing trend.

The primary objective of this study was to analyze the long‐term trends in childhood IRs of T1D over a 24‐year period in Lithuania and to investigate the seasonal patterns in the timing of the disease onset.

## 2. Methods

This is a retrospective study that focused on individuals aged 0–14 years diagnosed with T1D and registered in Lithuanian Children’s Diabetes Database. All patients with newly diagnosed T1D in Lithuania were included in the database during the period from 2001 to 2024. Lithuanian Children‘s Diabetes Database is a nationwide, population‐based database at the Department and Institute of Endocrinology, Lithuanian University of Health Sciences. This center serves as a diabetes reference center in Lithuania. All other pediatric endocrinology centers in Lithuania report new diagnoses of T1D in children under 18 years of age to this database. Population data required for this analysis were sourced from the Lithuanian Department of Statistics (https://www.hi.lt/).

Incidence data were collected following standardized protocols for comparable registries; the inclusion criteria were:

1. Diagnosis of T1D before the age of 15.
2. Confirmation of the T1D diagnosis according to International Society for Pediatric and Adolescent Diabetes (ISPAD) and/or American Diabetes Association (ADA), exact criteria depending on the year.
3. Permanent residency in Lithuania.

The study received Kaunas regional medical research committee permission (No. BE‐10‐6). The study was performed within the Declaration of Helsinki rules.

### 2.1. Statistical Analysis

Descriptive statistics were used to summarize the annual IRs of T1D over the 24‐year study period. Crude IR were calculated as the number of new T1D cases per 100,000 children aged 0–14 years in the population each year. These rates reflect the actual disease burden within the population without adjusting for age or sex. To describe annual crude incidence estimates of T1D, 95% Poisson confidence intervals were calculated for each year between 2001 and 2024. Given that annual case counts exceeded 20, an approximate method based on the normal approximation to the Poisson distribution was applied (the formula: CI_95%_ = ([*k* ± 1.96 × √*k*]/*n*) × 100,000), where *k* represents the number of new T1D cases and *n* the mid‐year population of children aged 0–14 years.

Temporal trends in rates from 2001 to 2024 were analyzed using joinpoint regression analysis. This approach identified points in time at which a statistically significant change in trend occurred and estimated the annual percent change (APC) for each segment, as well as the overall average APC (AAPC) across the study period.

Categorical and continuous variables were compared using appropriate statistical tests, with significance set at *p* < 0.05. All analyses were conducted using IBM SPSS Software version 30.0.

## 3. Results

### 3.1. Overall Trends: Annual IRs and Changes Over Time

Over the 24‐year study period, a total of 2369 newly diagnosed cases of T1D in children under the age of 15 were recorded, comprising 1181 boys and 1188 girls. The overall mean IR of T1D was 21.4 per 100,000 < 15 years (95% CI: 18.66–24.05).

**Figure 1:**
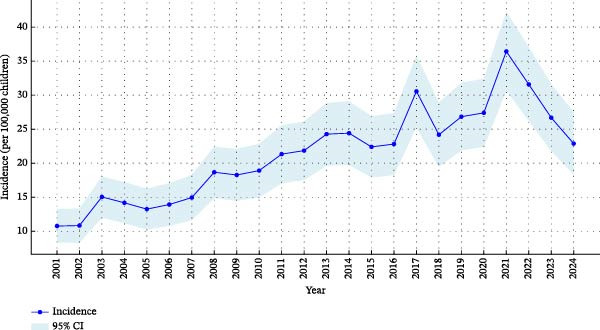
The incidence and ±95% Poisson CI in type 1 diabetes in children under the age of 15 during the period 2001–2024 (blue line).

The annual IR ranged from 10.8 to 36.4 per 100,000 < 15 years (Figure 1). The highest incidences were observed in 2021 and 2022, with a prior peak in 2017, whereas the lowest rates were recorded in 2001 and 2002.

**Figure 2:**
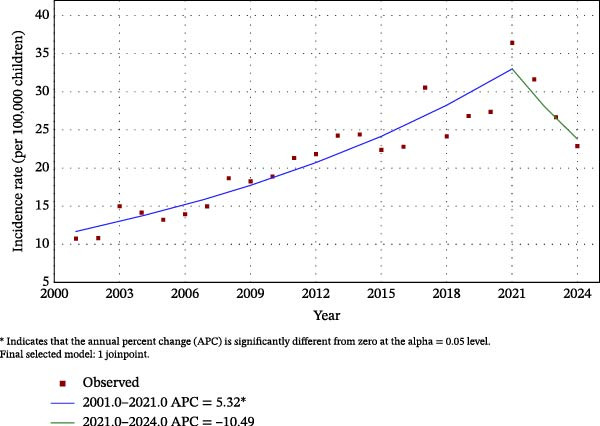
Joinpoint regression analysis of rates from 2001 to 2024. The model identified one statistically significant joinpoint in 2021. Solid line represent fitted trends, with APC values indicated for each segment.

Joinpoint regression analysis identified one statistically significant change in trend occurring in 2021. From 2001 to 2021, rates increased significantly, with an APC of 5.32% (*p*  < 0.001). From 2021 to 2024, a downward trend was observed; however, this decline did not reach statistical significance (APC: −10.49%, *p* = 0.086). Over the entire study period (2001–2024), the AAPC was 3.11% (95% CI: 1.35–4.65; *p* = 0.001), indicating a modest but statistically significant long‐term increase, presented in Figure 2.

### 3.2. Overall Trends: Subgroup Analysis

**Figure 3:**
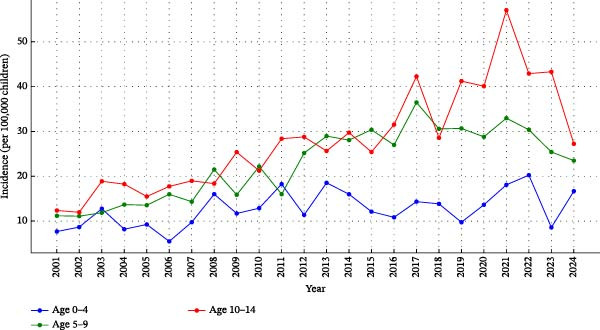
The incidence of type 1 diabetes in different age groups (blue 0–4, green 5–9, and red 10–14 years) from 2001 to 2024.

The mean IR of T1D in the youngest group (0–4 years) was 12.7 (95% CI: 11.1–14.3), in 5–9 years group 22.8 (95% CI: 19.6–25.9), and in 10–14 years group 28 (95% CI: 23.4–32.6), per 100,000 person‐years, *p*  < 0.0001. Post hoc analysis showed that the 5–9 years and 10–14 years age groups had higher IR than 0–4 years group, *p* = 0.0003 and *p*  < 0.0001, respectively. Comparison of the 5–9 years and 10–14 years age groups did not show significant difference, *p* = 0.084. The trends over 24‐year period are shown in Figure 3 for each age group.

The average yearly increase in T1D incidence from 2001 to 2024 in overall was + 3.3%, for each age group separately: 0–4 years + 5.1% per year; 5–9 years + 4.8% per year; 10–14 years + 5.2% per year; *p*  < 0.05. The pairwise comparisons between all age groups showed significant difference, *p*  < 0.001.

The mean IR of T1D in boys and girls were 16 (95% CI: 15.1–16.8) and 16.9 (95% CI: 16–17.8) per 100,000 < 15 years, respectively (z = −1.39, *p* = 0.165). Boys‐to‐girls mean ratio was 1.02.

### 3.3. Seasonal Variability

**Figure 4:**
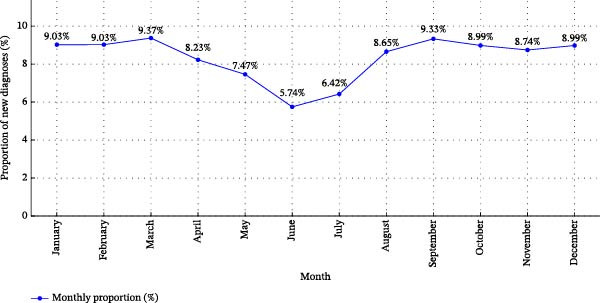
Monthly proportions of new type 1 diabetes cases in children during 2001–2024.

A significantly higher proportion of new T1D diagnoses was observed during the autumn–winter period (September to March, 63.5%) compared to the spring–summer period (April to August) (*t* = 3.21, *p* = 0.031). The distribution of new T1D cases by months is illustrated in Figure 4.

## 4. Discussion

This nationwide study, encompassing 2369 children and adolescents (aged 0–14 years) newly diagnosed with T1D over a 24‐year period in Lithuania, identified the highest incidence in 2021, reaching 36.4 cases per 100,000 < 15 years. Lithuania emerged as one of the leading European countries for new pediatric T1D cases, alongside its Baltic neighbors—possibly reflecting shared genetic backgrounds and environmental exposures [1, 2]. A key strength of this study is the comprehensive national coverage of pediatric T1D cases in Lithuania over a 24‐year period. Our findings highlight a need for comprehensive, longitudinal, and multidisciplinary research to elucidate the underlying the interplay of environmental, genetic, and societal factors contributing to the rising burden of T1D during childhood.

The IR of T1D in Lithuania peaked during the COVID‐19 pandemic, as defined by the World Health Organization (WHO) from early 2020 to late 2022/early 2023 [3]. This trend aligns with findings from studies in Germany [4] and two major systematic reviews and meta‐analyses conducted by Rahmati et al. [5] and D’Souza et al. [6], all authors reported a significant increase in pediatric T1D incidence when comparing the pre‐pandemic and pandemic periods. However, a notable limitation of our study—shared by many others [7–10]—is the lack of direct virological evidence, such as the presence of SARS‐CoV‐2 antigen or antibodies at or prior to diabetes onset, which prevents definitive conclusions about the causal role of the virus. Additionally, several studies have proposed that indirect consequences of the pandemic, particularly lockdown‐related factors, may have contributed to the observed rise in incidence [5–9, 11].

While changes in T1D incidence in some countries have been partially attributed to immigration—such as the observed impact of Asian immigration in Sweden [12]—this factor is unlikely to explain the trend in Lithuania. Immigration levels in Lithuania remain relatively low and are predominantly from neighboring countries like Ukraine and Belarus, with only sporadic cases originating from more distant regions, such as Asia or Africa, according European Migration Network (https://www.emn.lt/en/).

Another notable finding in our study was the seasonal variation in the incidence of T1D among children, which aligns with previous reports—particularly those from the Northern Hemisphere—demonstrating higher diagnosis rates during the darker months of the year (autumn and winter) [13–16]. One proposed explanation involves reduced sun exposure during these periods, leading to lower cutaneous synthesis of vitamin D. Given the immunomodulatory properties of vitamin D and its potential protective role in pancreatic β‐cell function, its deficiency may contribute to increased T1D susceptibility [14–16]. Additional hypotheses include seasonal fluctuations in viral infections, changes in school attendance patterns, and variations in physical activity, all of which may influence the risk of disease onset [13–17].

One potential limitation of our study is the possibility of diabetes misclassification. While type 2 diabetes (T2D) remains relatively uncommon in Lithuanian children, with up to 55 reported cases in 2024 (https://stat.hi.lt/), the distinction between T1D and T2D has been carefully made by pediatric endocrinologists. Since 2012, the routine use of pancreatic autoantibody testing has been implemented in all pediatric diabetes centers across Lithuania. Prior to that, T1D diagnoses were based on clinical features, C‐peptide levels, and the presence of absolute insulin deficiency. Additionally, it is important to acknowledge that before the widespread introduction of antibody and genetic testing, some cases of monogenic diabetes—though rare and accounting for up to 5% of all diabetes cases—may have been misclassified.

This study demonstrated a marked yearly increase in T1D incidence among children, with the most substantial rise observed in the early adolescent group (10–14 years). Similar patterns have been reported in recent data from Poland, suggesting the presence of shared predisposing factors contributing to this phenomenon across neighboring countries [18]. While some stabilization in T1D incidence has been observed in some parts of Europe—particularly in Ireland [19], some regions of Spain [20], and Sweden [12]—the majority European countries continue to report heterogeneous upward trends in incidence, especially among adolescents, underscoring the growing public health burden of T1D in this age group. From a broader perspective on autoimmune diseases, recent studies have reported rising IRs of conditions such as rheumatoid arthritis, multiple sclerosis, psoriasis, inflammatory bowel disease, and asthma among adolescents and young adults [21]. These trends underscore the importance of developing comprehensive research frameworks and preventive strategies targeting this age group, particularly as they undergo critical physiological, emotional, and psychosocial transition that may modulate immune system vulnerability.

## 5. Conclusions

Over the 24‐year period from 2001 to 2024, the incidence of T1D among children in Lithuania nearly tripled. The highest mean incidence was recorded during the COVID‐19 pandemic years, with early adolescents (10–14 years) representing the largest proportion of newly diagnosed cases. These findings underscore the urgent need for further investigation into both the direct and indirect mechanisms underlying the pathogenesis of T1D, as well as the development of targeted preventive strategies and informed public health policies.

## Author Contributions

**Ingrida Stankute:** conceptualization, writing – original draft. **Dale Marciulionyte:** conceptualization, data curation. **Rytas Ostrauskas, Aiste Cemerkaite, Gryte Leonaviciute, Robertas Kemezys, and Sigita Vainiene:** data curation. **Kastytis Smigelskas:** formal analysis. **Rasa Verkauskiene:** conceptualization, writing – review and editing.

## Funding

No funding was received for this manuscript.

## Disclosure

The abstract “Incidence Trends and Seasonal Variations of New T1D Diagnoses in Lithuanian Children during 2001–2022” will be presented as a Poster at Joint Congress of ESPE and ESE 2025 in Copenhagen, Denmark. After using OpenAI, the authors reviewed and edited the content as needed and took full responsibility for the content of the publication.

## Conflicts of Interest

The authors declare no conflicts of interest.

## Data Availability

The data that support the findings of this study are available from the corresponding author upon reasonable request.
